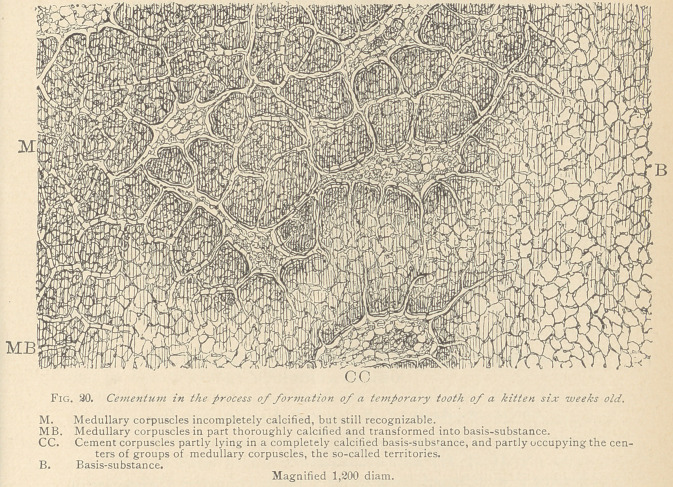# Contributions to the History of Development of the Teeth

**Published:** 1887-08

**Authors:** Carl Heitzmann, C. F. W. Bödecker


### Independent Practitioner.

VOL. VIII. AUGUST, 1887. NO. 8.

NOTE.—No paper published or to be published in another journal will be accepted for this department. All papers must be in the hands of the Editor before the first day of the month preceding that in which they are expected to appear. Extra copies will be furnished to each contributor of an accepted original article, and reprints, in pamphlet form, may be had at the cost of the paper, press-work and binding, if ordered when the manuscript is forwarded. The Editor and Publishers are not responsible for the opinions expressed by contributors. The journal is issued promptly. on the first day of each month.

### Original Communications.

### CONTRIBUTIONS TO THE HISTORY OF DEVELOPMENT OF THE TEETH.

BY CARL HEITZMANN, M. D., AND C. F. W. BÖDECKER, D. D. S., M. D. S.

CONTINUED FROM PAGE 349.

### III. DEVELOPMENT OF CEMENTUM.

Our researches on the history of the development of the teeth extend over a period of eight years, and a large collection of specimens has been at our disposal. Nevertheless, until recently, we hesitated about saying anything upon this topic, as none of the specimens of which we were then possessed showed anything of interest concerning the development of cementum. This tissue, in human beings, is obviously developed after birth, when the root and its dentine have been fully formed. We know that at the time of birth only the crowns of the temporary teeth are present, but no trace of the roots. At what time the tissue in question begins to appear in the human subject, the writers are unable to state. The only specimens at our disposal, in which the question of the development of the cementum could be studied, have been obtained from the lower jaw of a kitten about six weeks old. As the history of the development of dentine and enamel is almost identical in cats and men, we feel justified in the assumption that the formation of cementum is likewise identical. We are the more confident because of the knowledge that the development of bone exhibits the same features in cats as in men, and because, as is well known, cement is nothing but bone tissue.

In the above mentioned specimens from a kitten six weeks old, we observed both the temporary and permanent teeth in situation. The temporary teeth were fully developed and their roots perfect, whereas the permanent teeth only exhibited a small cap of dentine and enamel over the papilla, corresponding to a human fœtus that is between the seventh and eighth month of intra-uterine life. Both the dentine of the crown and that of the root, especially of the latter, were partly absorbed and exhibited bay-like excavations, which were filled with multinuclear protoplasmic masses.

The cement at the cervix of the teeth in cats is a comparatively narrow formation, made up, like that of men, of delicate spindles arranged vertically to the longitudinal axis of the root, between which we observe no cement corpuscles. This layer is evidently developed from spindle-shaped medullary corpuscles, of which a whole row is visible in the neighboring pericementum. A direct calcification of these medullary corpuscles leads to the formation of the cementum of the neck. Further down, the cementum becomes broader by the addition of a row of medullary corpuscles greatly varying in size, and without distinct lines of demarcation between them. With lower powers of the microscope their protoplasm appears finely granular; with high powers, however, a distinct reticulum is recognizable, the same as in all protoplasmic formations. Toward the dentine the medullary corpuscles assume a high grade of refraction, indicative of a deposition of lime-salts, which latter have been removed by the treatment with the chromic acid solution. The boundary line between the dentine and cementum is nowhere distinctly marked. Still further down toward the apex of the tooth, scanty cement corpuscles make their appearance, invariably surrounded by a number of finely granular medullary corpuscles, without any marked territorial formation around each bone corpuscle. Nearer to the apex the cementum becomes very broad, exhibiting a number of cement corpuscles. Here this tissue is fully developed around the dentine, but is yet in the process of formation at the periphery of the root. The latter portion plainly reveals the manner in which the cementum is developed.

FIG. 19. *Apex of the root of a temporary tooth of a kitten six weeks old. Vertical section.*

D. Dentine.

C. Cementum, in part thoroughly calcified, and partly made up of medullary corpuscles.

CB. Basis-substance of the cementum composed of medullary corpuscles.

PC. Pericementum.

Magnified 200 diam.

Like all the tissues of the body, including the dentine and the enamel, the cementum arises from medullary tissue. In this respect cementum and bone tissue show a striking coincidence. The medullary corpuscles from which bone tissue arises bear the name of osteoblasts, and should any one desire to give a special name to the formers of the cementum, that of cementoblasts should be admissible. These corpuscles become the seat of a deposition of lime-salts before any cement corpuscles are conspicuous. We observed that the previously calcified medullary corpuscles are decalcified, and after a second calcification some of them remained unchanged, exhibiting an angular shape characteristic of bone and cement corpuscles. This stage, however, still represents an incomplete form of cementum. Lastly, another decalcification of the medullary corpuscles takes place, and this time distinct groups of medullary corpuscles become visible, the centers of which are occupied by the cement corpuscles, and in this manner the territories of the cementum as well as those of bone tissue arise. After calcification of the medullary corpuscles has been accomplished, neither the medullary corpuscles nor the boundary lines of the territories are conspicuous; but when calcification is incomplete, both the medullary corpuscles and the territories are easily recognizable.

With high powers of the microscope we more readily observe the manner in which the cementum is developed. As long as this tissue is imperfect, and not fully calcified, a number of the medullary corpuscles assume a certain degree of refraction which designates partial calcification, whereas some of the medullary corpuscles retain their protoplasmic nature, and thus represent the cement corpuscles. Nearer to the dentine the medullary corpuscles are arranged in clusters, in the center of which we observe the cement corpuscles. As a rule, each cement corpuscle is surrounded by a number of medullary corpuscles representing a territory. Not infrequently a territory is indistinctly defined, and this is the case wherever the cement corpuscles are separated from each other by a single row of medullary corpuscles only, or where the deposition of lime-salts has been completed, when both the medullary corpuscles as well as the boundary lines of the territories, are lost to sight. (Fig. 20.)

The question arises, how are the offshoots of the cement corpuscles formed ? In order to understand this process, we must bear in mind that the medullary corpuscles, even when incompletely calcified, exhibit the reticular structure characteristic of all protoplasmic formations. They greatly vary in size and are separated from one another by light rims, which invariably appear traversed by delicate conical offshoots. It is obvious that the offshoots are formations of living matter, serving for the inter-connection of all medullary corpuscles. Whenever lime-salts are deposited in the meshes of the reticulum of the medullary corpuscles, the interstices between them remain free from such a deposit. The conical offshoots between the medullary corpuscles coalesce in their centers into a filament of living matter—the offshoot of the bone or cement corpuscle. Such a delicate offshoot remains inter-connected by the lateral filaments running into the reticulum of the medullary corpuscles, the same as is the case with dentine and enamel fibers. Even where the calcification of the medullary corpuscles has assumed its highest degree, and where all boundary lines between them have disappeared, the reticulum in the basis-substance remains unaltered, and plainly visible with a good immersion lens, without the addition of any re-agent.

The results of our researches concerning the history of development of the cementum of the temporary teeth of a kitten six weeks old are as follows :—

I. The cementum arises from medullary tissue, the same as bone and all other tissues of the body.

II. The medullary corpuscles first become the seat of a deposition of lime-salts before any cement corpuscles are visible.

III. The lime-salts are dissolved and re-deposited again in the medullary corpuscles, which are arranged irregularly around the cement corpuscles, the latter, however, remaining free from calcareous infiltration.

IV. Still later the medullary corpuscles are arranged in groups, the centers of which are the cement corpuscles, the sum total furnishing the territories around the cement corpuscles.

V. The offshoots of the cement corpuscles are filaments of living matter, originating from the bridges traversing the interstices between the medullary corpuscles.

VI. The reticular structure of the original medullary corpuscles is preserved in the basis substance even after the completion of the calcification of the latter, and the disappearance of the original medullary corpuscles.

(TO BE CONTINUED).

## Figures and Tables

**Fig. 19. f1:**
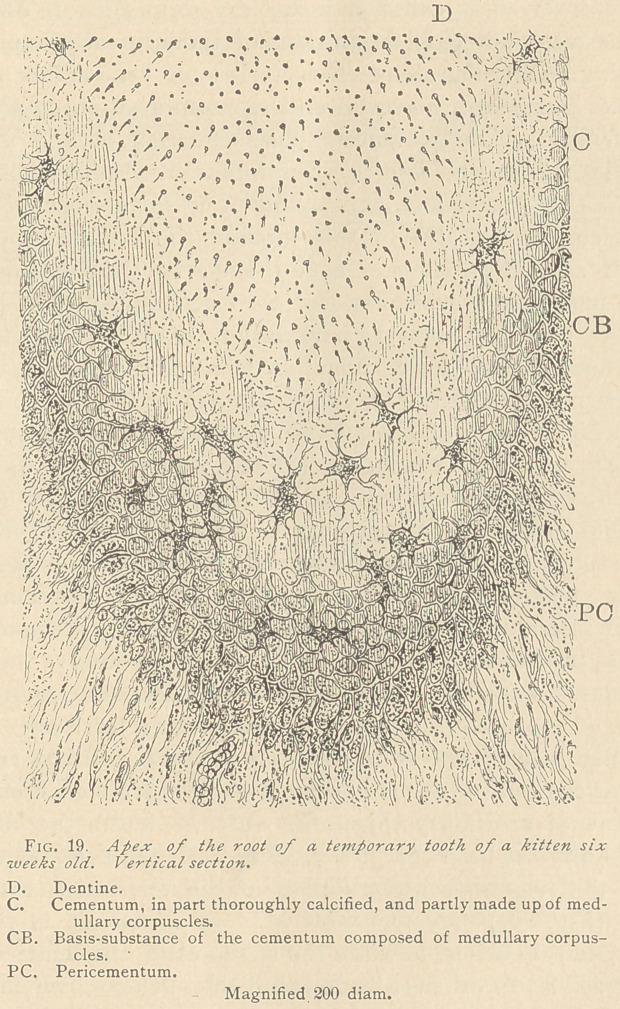


**Fig. 20. f2:**